# Lung Ultrasound Offers Fast and Reliable Exclusion of Heart Failure in the Emergency Department: A Prospective Diagnostic Study

**DOI:** 10.3390/diagnostics15243100

**Published:** 2025-12-06

**Authors:** Adis Keranović, Katja Kudrna Prašek, Ivan Gornik

**Affiliations:** 1 Department of Emergency Medicine, University Hospital Centre Zagreb, Kišpatićeva 12, 10000 Zagreb, Croatia; katja.kudrna.prasek@kbc-zagreb.hr (K.K.P.); ivan.gornik@kbc-zagreb.hr (I.G.); 2 School of Medicine, University of Zagreb, Šalata 3, 10000 Zagreb, Croatia

**Keywords:** lung ultrasound, acute heart failure, acute dyspnea, diagnostic accuracy, emergency department

## Abstract

**Background/Objectives**: Acute dyspnea is a common and urgent presentation in the emergency department, with acute heart failure (AHF) as one of its leading causes. Rapid differentiation between AHF and other etiologies is essential. **Methods**: This study aimed to evaluate the diagnostic accuracy of lung ultrasound (LUS) and compare it to chest X-ray (CXR) and NT-proBNP accuracy in patients with acute dyspnea, and to assess the potential of LUS for fast bedside diagnosis. This prospective study included 242 adult patients presenting with acute dyspnea of ≤3 days’ duration. All underwent NT-proBNP testing, CXR, and LUS according to a standardized protocol. The final diagnosis was established by experienced clinicians using all available clinical, laboratory, and imaging data, blinded to the LUS results. Diagnostic performance measures of LUS, CXR, and NT-proBNP were evaluated, and examination times of LUS and CXR were compared. **Results**: LUS achieved the highest sensitivity (95.3%) and negative predictive value (90.8%) for AHF, outperforming NT-proBNP (87.5%, 74.2%) and CXR (84.4%, 79.0%). CXR showed the highest specificity (65.8%) and positive predictive value (73.5%), while LUS specificity was moderate (51.8%). The LUS results were available significantly faster (median 10.0 min) than CXR (median 62.5 min). **Conclusions**: LUS demonstrated diagnostic accuracy comparable to CXR and NT-proBNP, with superior sensitivity, negative predictive value, and shorter time to results. These findings support its use as a rapid, non-invasive, first-line tool for excluding AHF in acute dyspnea patients.

## 1. Introduction

Acute dyspnea is among the most common and urgent reasons for emergency department (ED) visits and is frequently caused by acute heart failure (AHF), pneumonia, pulmonary embolism, or chronic obstructive pulmonary disease (COPD) [1,2]. Rapid and accurate differentiation of these conditions is critical to initiating appropriate therapy and improving patient outcomes [2]. The diagnostic challenge is intensified because these diseases often share overlapping clinical signs and symptoms, while diagnostic delays can have serious prognostic consequences. In patients with AHF, early identification and initiation of treatment are associated with reduced morbidity and mortality, whereas delays in diagnosis can lead to rapid clinical deterioration and prolonged hospitalization [3].

Chest radiography (CXR) and NT-proBNP measurement remain widely used tools in the evaluation of acute dyspnea. CXR, due to its broad availability and long-standing integration into emergency workflows, is typically the first imaging modality utilized. However, its sensitivity in early cardiogenic pulmonary edema is limited, and interpretation may be delayed, particularly in busy ED settings [4]. Furthermore, radiographic findings often appear only after significant pulmonary congestion has developed, which limits the ability of CXR to detect early or mild decompensation. Patient positioning, exposure quality, and reader experience also contribute to variability in diagnostic accuracy [5]. NT-proBNP is a valuable biomarker for heart failure diagnosis, reflecting myocardial wall stress and neurohormonal activation. Nevertheless, its specificity decreases in elderly patients and those with renal dysfunction, sepsis, or other comorbidities, which are common in the emergency population [6,7,8]. Elevated levels may therefore not always indicate a cardiac origin of dyspnea, and normal results cannot fully exclude heart failure in early or mild cases. Additionally, laboratory processing times can hinder rapid decision-making, particularly when immediate differentiation of cardiac and non-cardiac causes is required for triage and management.

In contrast, lung ultrasound (LUS) has emerged as a rapid, bedside, radiation-free technique that detects extravascular lung water through identification of B-lines—vertical hyperechoic artifacts arising from the pleural line [9,10]. B-lines correlate with pulmonary congestion and left ventricular filling pressures, providing real-time insights into hemodynamic status in suspected AHF [11].

Beyond its diagnostic capacity, LUS offers important advantages in patient monitoring and follow-up. Serial LUS examinations can quantify changes in pulmonary congestion in response to diuretic therapy, enabling individualized treatment adjustments and potentially preventing rehospitalization [12]. The method is also advantageous in terms of safety and logistics, eliminating radiation exposure and reducing the need for patient transport within the hospital, which are factors particularly relevant in the elderly and critically ill.

Previous studies suggest LUS may approach or exceed CXR and NT-proBNP diagnostic accuracy for AHF [13,14]. However, data come from heterogeneous populations with varying inclusion criteria, operator experience, and reference standards [15], limiting direct comparisons and emphasizing the need for standardized research designs in real-world emergency settings.

Although evidence supporting LUS has grown substantially, data directly comparing LUS with both CXR and NT-proBNP in the same cohort of patients presenting with acute dyspnea remain limited [13]. The relative diagnostic performance of these methods (and their impact on diagnostic efficiency) requires further clarification. Addressing this gap is particularly relevant in the modern ED, where timely and accurate differentiation between cardiac and non-cardiac causes of dyspnea is essential for optimizing patient care pathways and resource utilization.

Therefore, the aim of this study was to compare the diagnostic performance of lung ultrasound with that of chest X-ray and NT-proBNP in patients presenting with acute dyspnea in the emergency department. We also sought to evaluate the time required for each diagnostic method and to determine whether LUS can accelerate diagnostic decision-making while maintaining comparable accuracy. We hypothesized that lung ultrasound in patients with acute dyspnea provides diagnostic value equal to or superior to that of CXR and NT-proBNP, and that its rapid availability and bedside applicability make it a valuable first-line tool in the emergency evaluation of acute dyspnea.

## 2. Materials and Methods

### 2.1. Participants

This prospective study was conducted at the Emergency Department of the University Hospital Center Zagreb from February 2025 to May 2025 and enrolled adult patients (>18 years) presenting with acute dyspnea of ≤3 days’ duration in which AHF was considered a differential diagnosis.

Inclusion criteria were as follows: adult patients (>18 years), acute dyspnea as the leading complaint in the emergency department with heart failure as a possible cause (acute dyspnea was defined as new-onset or worsening shortness of breath lasting up to three days), patients who were conscious and breathing spontaneously, patients not requiring mechanical ventilation support, and having signed informed consent.

**Exclusion criteria were as follows**: unconscious patients, patients requiring mechanical ventilation (e.g., non-invasive ventilation), hemodynamically unstable patients requiring immediate resuscitation, patients requiring dialysis, chest trauma, or refusal to participate in the study. Patients with obvious causes of dyspnea (i.e., airway obstruction) were also excluded.

After meeting all inclusion criteria and completing the informed consent procedure, patients underwent further diagnostic evaluation. This included clinical examination, blood sampling for NT-proBNP analysis, and chest X-ray. These assessments resulted in a final clinical diagnosis of acute heart failure or an alternative condition. The overall clinical assessment—excluding lung ultrasound—served as the reference standard against which lung ultrasound results were later compared. Demographic data were obtained from previous medical records and the hospital information system.

### 2.2. Data Collection and Clinical Assessment

NT-proBNP measurement was performed at the Department of Emergency Laboratory Diagnostics, University Hospital Center Zagreb, using Roche Elecsys and Cobas e immunoassay analyzers. For patients younger than 50 years, a value > 450 ng/L was diagnostic for AHF; for those aged 50–75 years, >900 ng/L; and for patients over 75 years, >1800 ng/L. Values below 300 ng/L excluded AHF, while intermediate results were considered inconclusive.

Chest X-rays of the heart and lungs were performed at the Department of Diagnostic and Interventional Radiology, University Hospital Center Zagreb, in upright posteroanterior and lateral projections or supine anteroposterior, as per the patient’s condition. Patients were referred to the radiology department for chest X-rays upon the lead physician’s request, execution of which depended on the immediate availability of porters and the Radiology department workload.

Echocardiography was performed upon request of the lead physician when accessible. The results were not available to the LUS investigator but were included in the reference standard evaluation.

Radiographs were interpreted by a board-certified radiologist according to predefined criteria. A positive finding for AHF was defined by evidence of pulmonary venous redistribution, Kerley B lines, and interstitial or alveolar edema. A negative result indicated the absence of these features. The radiologist was blinded to the results of the lung ultrasound.

Lung ultrasound was performed immediately after blood sampling. As described in the introduction, the thorax was scanned in eight zones with the patient in a supine or sitting position, depending on clinical stability. The ultrasound devices used were Esaote MyLab Six CrystaLine ultrasound system (Esaote S.p.A., Genoa, Italy) (AC 2541 convex probe, 1–8 MHz) and Philips Affiniti 70 ultrasound (Bothell, WA, USA) (C5-1 convex probe, 1–5 MHz). Examination settings were the same on both machines: frequency 4 MHz, depth 15 cm, gain 50%, focal point set at the pleural line, dynamic range set low to high (wide). The standard BLUE protocol was used for thoracic ultrasound examination. Pleural sliding, A-profile, and B-profile were documented, as well as the presence of pleural effusion or other sonographic findings.

The examination began with rapid bilateral anterior orientation in two points, followed by systematic scanning of all eight zones. This included upper and lower anterior regions and upper and basal lateral regions on both hemithoraces. The primary investigator was a trained emergency medicine specialist and instructor in lung ultrasound; he performed all lung ultrasound examinations. In uncertain cases or unclear findings, a senior examiner independently repeated the scan. Uncertain and discrepant cases were labeled as inconclusive and excluded from statistical analysis. The investigator’s and the supervisor’s performance was validated prior to this study in a regular quality-assurance analysis in the Department, which showed no significant interobserver differences.

A positive lung ultrasound finding for lung congestion (suspected AHF) was defined as the presence of two or more positive zones with three or more B-lines per lung. Unilateral finding of B-lines, and findings of B-lines exclusively in apical segments of the lungs were labeled as “inconclusive” and not positive for congestion.

To compare diagnostic values of LUS, CXR, and NT-proBNP, the reference standard for AHF diagnosis was established through a structured, independent clinical assessment by an investigator blinded to LUS findings. Available clinical data were reviewed for each patient, including the following: clinical presentation (symptoms and physical examination findings), NT-proBNP levels interpreted using age-adjusted thresholds, chest radiography findings, response to diuretic therapy during ED stay or hospitalization, hospital course and discharge diagnosis, and echocardiographic findings when available.

A diagnosis of AHF required as follows: (1) clinical signs and symptoms consistent with acute heart failure; either (2) NT-proBNP elevation above age-adjusted threshold OR radiographic evidence of pulmonary congestion; or (3) clinical improvement with diuretic therapy. This reference standard was used for all sensitivity/specificity calculations.

For all patients, the total examination time was recorded, including the start of clinical evaluation, duration of the lung ultrasound procedure, and time of chest X-ray report completion.

### 2.3. Statistical Analysis

Quantitative data (age, NT-proBNP levels, examination times) were expressed as medians and quartile ranges. Differences in NT-proBNP by age groups were analyzed using the Kruskal–Wallis ANOVA. Sensitivity, specificity, positive predictive value (PPV), and negative predictive value (NPV) were calculated for NT-proBNP, CXR, and LUS using clinical diagnosis as the reference. Examination times were recorded and compared between LUS and CXR. Statistical significance was set at *p* < 0.05. Sample size requirements were calculated using an online calculator provided by Akoglu [16] with the following parameters: disease prevalence of 30%, α = 0.05, sensitivity of 90%, and marginal error of 10%. With these parameters, the required sample size was 115, based on which we conservatively determined the inclusion period of four months. The inclusion period was from February 2025 to May 2025.

## 3. Results

**Figure 1:**
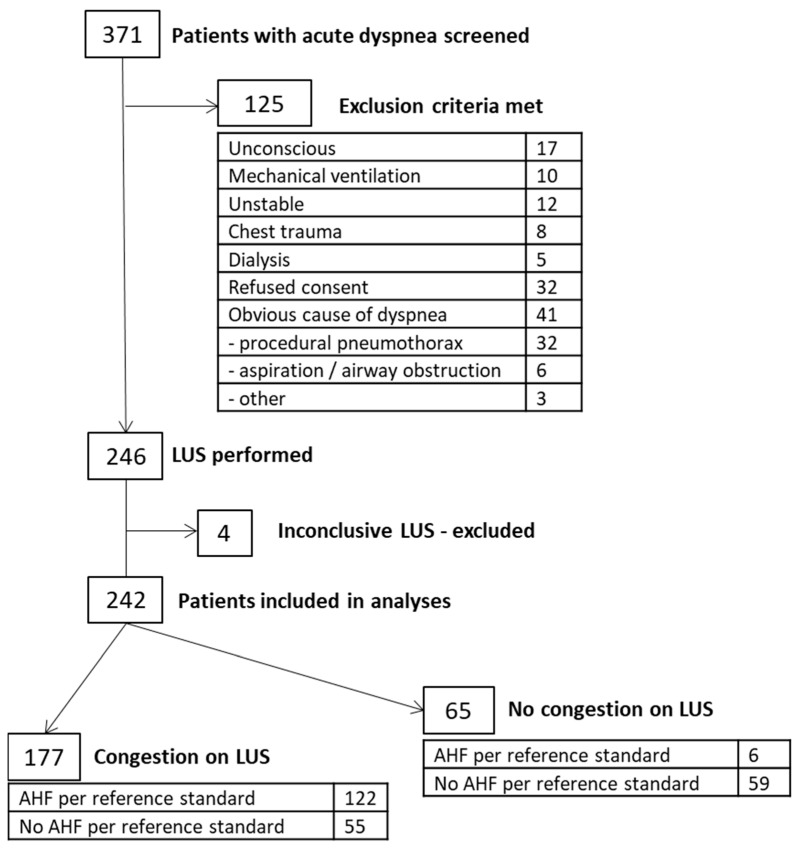
Flow and shedding of patients from screening to final diagnosis; LUS—lung ultrasound, AHF—acute heart failure.

During the inclusion period, 371 patients with an acute-onset of dyspnea were screened for inclusion. The patient flow is presented in Figure 1. Ultrasound was performed in 246 patients, of whom 4 were excluded due to inconclusive reports. The median age of the cohort was 79 years (range 30–100). Females were more frequent in this cohort (133 females, median age 80; 109 males, median age 76). Median ages were similar in patients with (80 years) and without AHF (77 years).

Ultrasound findings consistent with acute heart failure (congestion) were found in 177 patients, and negative findings were found in 65 patients. The final diagnosis of heart failure (per reference standard) was set in 128 patients (Table 1). Only a minority of patients (N = 35) were not admitted to the cardiology ward due to overcrowding during the study’s duration. For all admitted patients, the diagnosis of AHF was confirmed with echocardiography.

Table 2 presents statistics of NT-proBNP concentrations. The median NT-proBNP level in the study population was 3742.0 pg/mL. The lowest median NT-proBNP concentration was observed in patients younger than 50 years, while the highest median was recorded in patients older than 75 years. Differences in NT-proBNP levels across age groups were statistically significant (*p* < 0.05). The interquartile ranges of NT-proBNP concentrations were wide both in the overall population and across all age categories, indicating substantial variability.

LUS demonstrated the highest sensitivity (95.3%) and NPV (90.8%) in diagnosing AHF, exceeding NT-proBNP (87.5% sensitivity, 74.2% NPV) and CXR (84.4% sensitivity, 79.0% NPV). CXR had superior specificity (65.8%) and PPV (73.5%) compared to LUS (51.8% specificity, 68.9% PPV) and NT-proBNP (40.4% specificity, 62.2% PPV) (Table 3).

**Figure 2:**
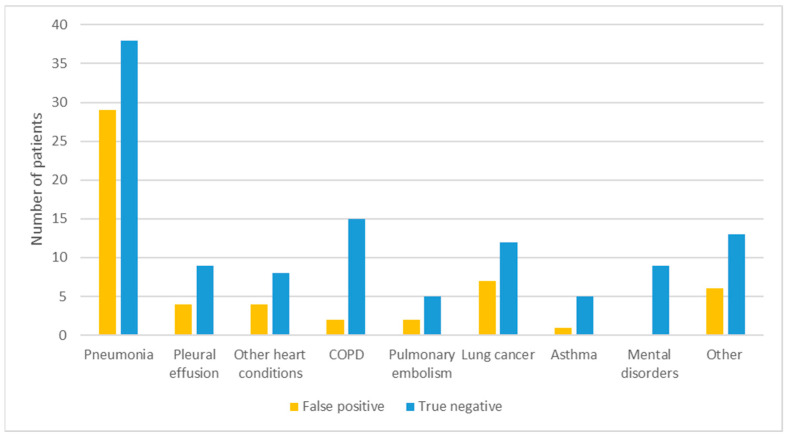
The number of false-positive reports (ultrasounds reported as congestions) relative to true negatives by final diagnosis. COPD—chronic obstructive pulmonary disease.

Analysis of ultrasound findings that were false positives for congestion (Figure 2) revealed that more than 50% of those were, in fact, due to pneumonia and lung cancer (as per the reference standard). Pleural effusions and other cardiac diseases were misclassified as AHF in approximately 50% of cases. False-positive AHF diagnoses were less frequent in patients with COPD and asthma.

**Figure 3:**
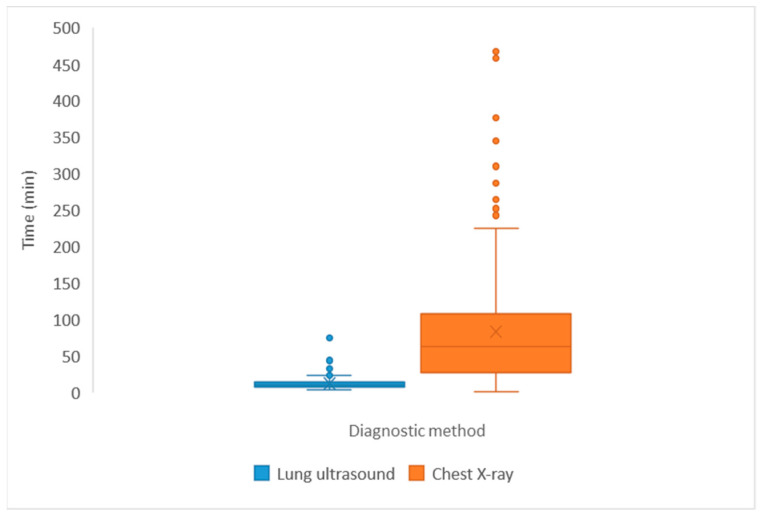
Comparison of times to obtain lung ultrasound and chest X-ray report (*p* < 0.05).

The time to ultrasound findings showed a relatively narrow distribution, ranging from 4.0 to 75.0 min, with a median of 10.0 min and a quartile range from 8.0 to 14.0 min. The median waiting time for chest X-ray results was 62.5 (28.25–106.5) min, with the longest waiting time recorded at 474.0 min. All LUS results preceded CXR reports (Figure 3).

## 4. Discussion

The main finding of our study is that lung ultrasound (LUS) achieved the highest sensitivity (95.31%) and negative predictive value (90.77%) among the evaluated diagnostic methods for acute dyspnea, exceeding the corresponding values for NT-proBNP (87.50% and 74.19%) and chest X-ray (84.38% and 78.96%). Chest X-ray, however, demonstrated the highest specificity (65.79%) and positive predictive value (73.47%), while LUS showed moderate specificity (51.75%) and NT-proBNP the lowest (40.35%). These results suggest that LUS is particularly effective for ruling out acute heart failure (AHF) but may be less reliable for confirming the diagnosis compared with chest X-ray.

Taken together, our findings partially affirm the initial hypothesis. LUS matched or exceeded chest X-ray and NT-proBNP in sensitivity and NPV, but did not outperform chest X-ray in specificity or PPV. While LUS provided results much faster than chest X-ray and was valuable for follow-up assessment of treatment response in AHF, its lower specificity (51.75% vs. 65.79%) indicates a greater potential for false-positive findings.

The median age of the study population was 79.0 years, placing this research among those with a high patient age. Glöckner et al. [14] reported a median age of 72 years in a similar study, Conangla et al. [17] reported 75.6 years, and Sartini et al. [13] reported 78 years. Furthermore, in our study, the age of patients with acute heart failure and those without the condition was very similar, making them suitable for comparative analysis.

In diagnostic test evaluation, sensitivity, specificity, and predictive values are calculated against a reference standard [18]. Echocardiography is considered the reference standard for lung ultrasound, but it is often unavailable in emergencies. Alternatives include expert panel diagnosis or NT-proBNP concentration, with age-adjusted cut-offs [19,20,21,22,23,24]. NT-proBNP is highly sensitive but less specific, as elevated levels occur in other conditions such as pulmonary embolism and atrial fibrillation [25,26,27]. In our study, the median values of NT-proBNP increased with age and the prevalence of acute heart failure, reflecting the known age dependence [28,29].

In our study, LUS demonstrated very high sensitivity in diagnosing AHF, despite the study population having a relatively advanced median age. Sartini et al. [13] reported lower LUS sensitivity (57.73%) and chest X-ray sensitivity (74.99%) in a similarly aged cohort. One key difference is that Sartini’s team used a six-zone ultrasound protocol, while we applied the internationally recommended eight-zone approach [30], which may improve detection accuracy. Moreover, Sartini et al. noted a high proportion of patients who received diuretics before imaging, a factor shown by Volpicelli et al. [30] and others [31,32,33] to reduce the visibility of diagnostic B-lines, thereby lowering sensitivity. Glöckner et al. [14] also observed lower LUS sensitivity (75%) in an older population with comorbidities, attributing this partly to delayed ultrasound timing (average 60 min after admission versus 14 min in our study) and diuretic use. Pivetta et al. [34] reported similarly high sensitivity (97%) by using strict ultrasound criteria, reinforcing the concept that protocol and timing significantly impact diagnostic performance. Sartini et al. [13] reported a high sensitivity of NT-proBNP (97.59%) and a low specificity (27.56%). According to the authors, NT-proBNP is useful primarily for ruling out the possibility that a patient has acute heart failure.

The specificity of LUS recorded in our research was low, limiting its clinical use in diagnosing AHF. This low specificity is partly due to B-lines not being pathognomonic for AHF, as they also occur in other lung diseases such as pneumonia and fibrosis [35], and older individuals naturally have more B-lines [36]. Chest X-ray showed somewhat higher specificity (65.79%), with the highest specificity for the finding of alveolar edema, indicating the reliability of this radiological sign [15]. Sartini et al. [13] reported significantly higher specificity for lung ultrasound (87.97%) and chest X-ray (86.26%), while Glöckner et al. [14] reported 97.5% specificity in patients not treated with diuretics. Pivetta [34] and Buessler [37] also reported specificities around 97%, and Conangla et al. [17] found specificities up to 99% using two different LUS methods.

In our research, LUS showed a high negative predictive value (NPV) in excluding acute heart failure, which is crucial given the condition’s severity. Compared to chest X-ray and NT-proBNP, LUS demonstrated comparable or superior diagnostic utility. Other studies report varied results: Glöckner et al. [14] found a high positive predictive value (PPV) but a moderate NPV for LUS, improving after excluding patients on diuretics. Pivetta et al. [34] reported very high PPV and NPV, likely due to strict criteria reducing false positives. Sartini et al. [13] observed moderate PPV and NPV for LUS, with chest X-ray performing slightly better, though both methods showed limitations due to false positives and negatives. Conangla et al. [17] demonstrated very high PPV and NPV for one LUS protocol (LUS-C2) but did not explain the cause. Overall, while all three methods (LUS, chest X-ray, and NT-proBNP) have diagnostic value, LUS stands out for its practical role in quickly and reliably ruling out acute heart failure, especially in urgent care settings.

A key strength of LUS is its rapid availability. In our study, the time to results was significantly shorter than for chest X-ray (median 10.0 vs. 62.5 min), supporting its role as a first-line test in acute dyspnea. The clinical importance of speed should not be underestimated. Rapid diagnosis facilitates the initiation of earlier treatment, which may improve outcomes. For example, point-of-care LUS has been shown to alter diagnoses in a substantial proportion of patients, directly influencing management decisions [38]. Moreover, studies report that LUS use improves patient flow and resource utilization in emergency departments [39], reduces delays in critical care linked to higher mortality [40], and contributes to shorter length of stay and better survival outcomes [41].

Beyond these operational advantages, the diagnostic immediacy of LUS carries important implications for clinical decision-making. In real-world emergency settings, where time is critical, the ability to obtain immediate, reproducible, and bedside imaging findings provides a significant advantage over conventional radiographic modalities. This speed allows physicians to differentiate cardiac from non-cardiac causes of dyspnea within minutes, guiding therapy earlier and potentially preventing disease progression. Furthermore, minimizing patient transport for imaging not only reduces the logistical burden on staff but also lowers the risk of deterioration in unstable patients. Taken together, these benefits reinforce the role of LUS as not only an accurate but also a time-efficient and patient-centered diagnostic modality, whose implementation may meaningfully improve both workflow efficiency and clinical outcomes.

## 5. Limitations

This study has several limitations. It was conducted in a single tertiary care center, which may limit generalizability to other emergency settings with different patient populations and resources. Lung ultrasound was performed primarily by a single investigator, and interobserver variability was not systematically assessed, which raises the possibility of operator-dependent bias. Exclusion of inconclusive cases from the analysis could also have influenced the results’ validity, but only four patients were excluded based on this. The small number of inconclusive cases also illustrates the method’s value.

Non-simultaneous LUS investigation and chest X-ray acquisition may have influenced the results, allowing any interventions to lessen the congestion identifiable by X-ray. Since the delay was not that pronounced in most patients, we feel it did not markedly influence results.

The reference standard diagnosis relied on a composite of clinical, laboratory, and radiological findings rather than echocardiography, which may have influenced the calculated diagnostic accuracy of the evaluated methods. The primary goal was to investigate the role of LUS in differentiating patients with heart failure among the population of patients with dyspnea, not to investigate cardiac performance in detail; echocardiography was requested by lead physicians according to their needs and understanding of a patient, and when available, was included in the reference standard evaluation. Echocardiography is the pivotal diagnostic tool in heart failure, but its availability in the emergency department is scarce, especially in unselected dyspnea patients.

Additionally, comorbid conditions such as pneumonia, chronic obstructive pulmonary disease, and lung cancer contributed to false-positive ultrasound findings and reduced specificity. The advanced age of the study cohort may also have affected NT-proBNP levels and the prevalence of B-lines unrelated to acute heart failure.

Follow-up imaging was limited to repeat lung ultrasound in selected patients, restricting conclusions about the comparative value of different modalities for monitoring treatment response. Finally, examination and reporting times may vary between institutions; therefore, the time advantage of ultrasound over chest radiography observed here may not be fully reproducible.

## 6. Conclusions

The findings of this study suggest that lung ultrasound is a valuable tool for evaluating patients with acute dyspnea, especially for differentiating patients without lung congestion due to AHF, thereby targeting further diagnostic efforts away from AHF. Its diagnostic accuracy, namely sensitivity and negative predictive value, is high, which makes it, combined with its simplicity, non-invasiveness, and markedly shorter examination time, one of the tools of choice for ruling out acute heart failure. The significantly reduced time to diagnosis compared with chest radiography supports the premise that lung ultrasound offers a time-efficient and clinically valuable alternative, particularly in emergency care environments where rapid decision-making is critical.

## Figures and Tables

**Table 1:** Confusion matrix for LUS against the reference standard.

	AHF Present *	AHF Absent *	Total
LUS positive **	122	55	177
LUS negative **	6	59	65
Total	128	114	242

LUS—lung ultrasound, AHF—acute heart failure; * as per reference standard; ** positive or negative for congestion, suggesting acute heart failure.

**Table 2:** NT-proBNP levels in the study population.

Age	NT-proBNP (pg/mL)		
	*N*	Median	Lower–Upper Quartile
All	242	3742.0	1279.0–12,092.0
<50 years	17	167.0 ^a^	83.0–851.0
50–75 years	74	2759.0 ^b^	1035.0–9219.0
>75 years	151	5720.0 ^c^	1966.0–13,684.0

^a,b,c^—Median values marked with different letters differ significantly at the *p* < 0.05 level.

**Table 3:** Diagnostic performance of lung ultrasound, chest X-ray, and NT-proBNP as tests for acute heart failure.

Test	Diagnostic Value Indicator			
	Sensitivity % (CI 95%)	Specificity % (CI 95%)	PPV % (CI 95%)	NPV % (CI 95%)
LUS	95.31 (90.08–98.26)	51.75 (42.20–61.21)	68.93 (64.63–72.92)	90.77 (81.53–95.63)
NT-proBNP	87.50 (80.50–92.68)	40.35 (31.27–49.95)	62.22 (58.28–66.01)	74.19 (63.33–82.72)
Chest X-ray	84.38 (76.91–90.19)	65.79 (56.32–74.42)	73.47 (67.99–78.31)	78.95 (71.05–85.14)

LUS—lung ultrasound, PPV—positive predictive value, NPV—negative predictive value.

## Data Availability

The original contributions presented in this study are included in the article. Further inquiries can be directed to the corresponding author.

## Abbreviations

The following abbreviations are used in this manuscript:

ED	Emergency department
LS	Lung ultrasound
NT-proBNP	N-terminal pro-B-type natriuretic peptide
CXR	Chest X-ray
PPV	Positive predictive value
NPV	Negative predictive value
AHF	Acute heart failure
COPD	Chronic obstructive pulmonary disease
